# Ureteral Occlusion: Device Strategies, Approaches, and Results

**DOI:** 10.1155/aiu/7843401

**Published:** 2025-07-02

**Authors:** Benjamin Treutler, Sahana Kumar, Christopher Shallal, Aryaman Gupta, Sanjana Kumar, Nicholas Zhang, Sean Healy, Jayaram Mandavilli, Nehali Gupta, Elizabeth A. Logsdon, Jordan Shuff, E. James Wright, Clifford R. Weiss

**Affiliations:** ^1^Department of Biomedical Engineering, The Johns Hopkins University, Baltimore, Maryland, USA; ^2^Department of Radiology and Radiological Science, The Johns Hopkins University, Baltimore, Maryland, USA; ^3^Center for Bioengineering, Innovation, and Design, The Johns Hopkins University, Baltimore, Maryland, USA; ^4^Department of Urology, The Johns Hopkins University, Baltimore, Maryland, USA

## Abstract

Genitourinary tract injuries can occur in the urinary tract or reproductive system as a result of trauma-related pelvic fractures, iatrogenic lacerations or ligations, and radiation therapy for reproductive or digestive malignancies. Although surgical reintervention is possible for large urinary tract injuries, a key component for healing smaller injuries is the ability to divert urine from the injury site to prevent urine-wound contact. This enables the injury to heal prior to reintervention and can eliminate the need for a secondary procedure, reducing the potential for complications. This type of urinary diversion is required by 140,000 patients in the United States annually, leading to the development of several devices to divert urine. The current standard of care includes minimally invasive procedures, such as placement of a catheter, double-J stent, or nephroureteral stent, but such measures often do not maintain sufficient dryness to enable wound healing. Based on a review of the literature, we have determined that successful devices need to prevent 100% of the anterograde urine flow, resist migration down the ureter because of peristalsis, and prevent urothelium growth over the device to promote wound healing without causing complications or necessitating reintervention. We also evaluated these devices according to the robustness of the study populations and designs in which they are reported. Some of the more successful devices include detachable, semicompliant balloons, platinum coils, and ureteral clips. Here, we present a narrative review of temporary and permanent ureteral occlusion devices and evaluate their potential for supporting wound healing. We also explore metrics by which to compare and select appropriate devices for urinary diversion.

## 1. Introduction

### 1.1. Types of Urinary Tract Injuries

Urinary tract injuries can be the result of pelvic trauma, iatrogenic laceration, ligation, devascularization, or pelvic radiation therapy. Common iatrogenic urinary tract injuries include those of the renal parenchyma, renal vasculature, ureter, and bladder [1]. These injuries can occur in all anatomical structures within the urinary system, resulting in urinary fistulae, stricture, and leakage [2, 3]. The most common iatrogenic renal injuries are vascular. These injuries happen during various surgical and endourological procedures, including percutaneous renal biopsy, percutaneous nephrostomy (PCN), percutaneous nephrolithotomy, endopyelotomy, and partial nephrectomy. Iatrogenic injuries to renal allografts after transplantation, though not discussed in this review, are more common and may even include arterial dissection [1]. Although intraoperative recognition of the injury with retrograde pyelography or ureteroscopy leads to the best outcomes, only one-third of iatrogenic ureteral injuries are identified during the procedure [1]. Common postoperative symptoms include pyrexia, hematuria, dysuria, and peritonitis with leukocytosis [1]. In the context of recent abdominopelvic surgery, urinary tract injury should be suspected and investigated [4].

If a genitourinary tract injury is present, urine leakage can occur through the wound site, impeding wound healing [5] and causing urine accumulation in the retroperitoneal space. This leads to pain, urinary tract infection, and urosepsis [6]. Urine-wound contact leads to tissue degradation, urokinase-mediated blood clot degradation, impeded wound closure, and infection [7]. To mitigate these effects, urinary diversion is often necessary.

### 1.2. Diagnosis of Urinary Tract Injury

Historically, intravenous urography has been used to diagnose missed ureteral injury. However, CT urography has replaced intravenous urography, and triple-phase contrast-enhanced CT with nephrographic and excretory phases offers greater sensitivity and specificity. Concerning radiographic signs include contrast extravasation and fluid collections suggesting urinoma. If uncertainty remains after CT urography, then bilateral retrograde pyelography should be performed [1].

### 1.3. Urinary System Diversion

**Figure 1:**
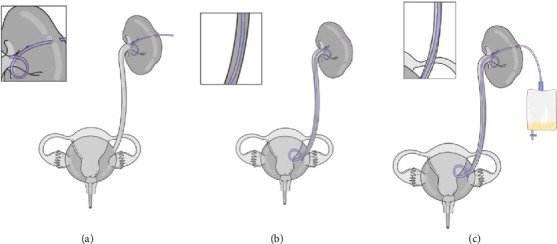
(a) Percutaneous nephrostomy tube in a urinary system blocked by a kidney stone. (b) Double-J ureteral stent. (c) Nephroureteral stent in a healthy urinary system.

Treatment of upper urinary tract injury involves placement of a retrograde ureteral stent or PCN tube (Figure 1(a)) that can enable wound healing without more invasive procedures, such as urinary diversion or suturing the wound closed [8, 9]. In more severe cases, in which there is no physiological obstruction in the urinary system, stents and catheters are deployed to create a bypass that diverts urine from the kidney directly into the bladder [10].

Recently, focus has shifted to minimally invasive techniques to achieve urinary diversion, such as placement of a catheter, double-J stent (Figure 1(b)), or nephroureteral stent (Figure 1(c)) [1]. Reported success rates of catheterization in urinary tract fistulae are 20%–75% [11], and incomplete diversions necessitate surgical reintervention to prevent further urine-wound contact and to facilitate healing [8, 12, 13]. Ureteral stents have also been shown to be ineffective, with some patients experiencing insufficient drainage and impaired renal function [8, 9]. Leakage after stent removal is common and requires additional surgical treatment.

Here, we review various catheter-based techniques for temporary and permanent urinary diversion in patients with urine leakage. Their effectiveness is evaluated by (1) ability to be deployed/removed from the ureter, (2) anchoring against the migratory peristaltic forces of the urinary system, (3) seal effectiveness, and (4) rate of complications and failures. While effectiveness is a critical factor in selecting a diversion technique, real-world use is also dependent on technique cost and patient-specific factors such as anatomy and life expectancy.

## 2. Unobstructed Ureteral Injury Requiring Diversion

### 2.1. Iatrogenic Urinary Tract Injury

Seventy-five percent of ureteral injuries are iatrogenic because of the ureters' proximity to the surrounding vasculature and because the ureters run along every level of the retroperitoneal and upper pelvic spaces, making it easy for accidental surgical complications to occur [1, 8, 14]. Common mechanisms include inadvertent ligation during laparoscopic gynecological procedures, ureteral perforation during ureteroscopy, and obstruction by sutures [1]. Approximately 90% of these injuries occur in the distal ureter [1, 14] because the proximal ureter is involved in fewer procedures and is more difficult to access; proximal ureter injury is commonly seen in noniatrogenic ureteric trauma. For small iatrogenic wounds, clinicians often attempt minimally invasive treatment, namely percutaneous urinary diversion.

### 2.2. Urinary Fistulae

Urinary fistulae can involve any part of the urinary tract, including kidneys, ureters, bladder, or urethra. Common examples include vesicovaginal, ureterovaginal, urethrovaginal, vesicouterine, colovesical, and ureteroarterial fistulae. Lower ureteric fistulae can be categorized as ureterovaginal or ureteroenteric fistulae. Most ureterovaginal fistulae are caused by pelvic surgery such as hysterectomies (54%) or obstetric complications such as birth trauma. Ureteroenteric fistulae are commonly caused by colorectal malignancy, radiation therapy, pelvic surgery, or inflammatory bowel disease [8].

The reported incidence of ureteral injury during gynecologic surgery depends on whether the surgery is to treat benign disease (1%) or malignancy (5%) [8]. One study reported that after hysterectomy for benign indications in nearly 300,000 women, the rate of urologic injury was 1.9%; of these injuries, 15% were fistulae [15]. It is typically recommended to wait at least 3 months before surgical repair to allow inflammation to subside, avoiding further damage from the secondary procedure [8]. Percutaneous urinary diversion is used to help reduce inflammation, which often also helps heal smaller or less complicated fistulae, eliminating the need for surgery.

Unfortunately, this combination of nonoperative and surgical fistula treatment fails in nearly 35% of patients [8]. To prevent further damage, retrograde ureteral stents or PCN tubes can be used to decrease urine flow around the injured area. This approach is often sufficient for small fistulae, but for large, unresponsive fistulae, other forms of urinary diversion or fistula closure such as permanent occlusion, plugging, or surgical repair are often necessary to alleviate symptoms and allow wound healing.

Urinomas secondary to urinary fistulae are rare and often reabsorb without intervention; however, in cases of expanding urinomas, they must be percutaneously drained with a catheter, in addition to empirical treatment with antibiotics. If a drainage catheter is inadequate, a PCN tube with a ureteral stent may be placed to promote healing. The third-line treatment includes surgical reconstruction [16].

## 3. Complete, Permanent Occlusion Solutions

Although there have been few recent attempts at permanent occlusion because of the popularity of PCN tubes among interventional radiologists, some efforts have been made to occlude the ureter in a novel manner. Indications for permanent occlusion include treatment-refractory urinary leaks, high pelvic tumor burden, prior radiation therapy, or patients who are poor surgical candidates because of comorbidities [17]. Occlusion can decrease pain, improve quality of life, and reduce the economic burden caused by poor wound healing and repeated need for reintervention [18]. Below, we summarize a series of permanent occlusion solutions with various degrees of success that have been developed during the past 30 years. We evaluate the clinical context of device use, as well as the iterative evolution of these devices to understand the patient care strategies for various ureteral diseases.

### 3.1. Transrenal Occlusion With Detachable Balloons

**Figure 2:**
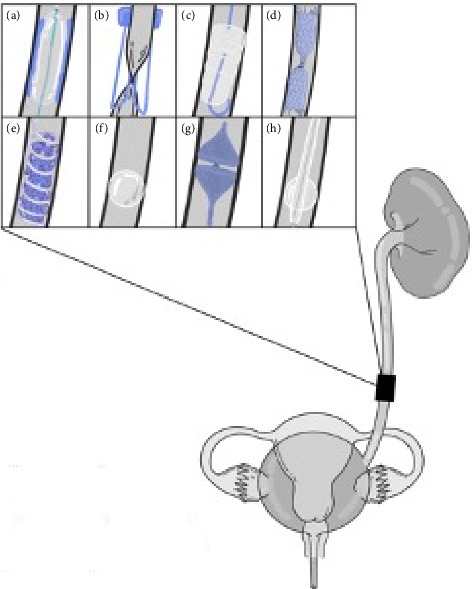
(a) Transrenal occlusion with a detachable balloon and butyl-2-cyanoacrylate. (b) Ureteral occlusion via clipping. (c) Ureteral occlusion via a silicone plug (Harzmann Olive technique). (d) Transrenal occlusion with crimped stents. (e) Platinum coil embolization with gelfoam pledgets. (f) Temporary ureteral occlusion via detachable, semicompliant balloons. (g) Ureteral occlusion via Amplatzer Vascular Plug II (Abbott, Plymouth, MN). (h) Temporary ureteral occlusion via reversible balloon nephrostomy.

To investigate complete diversion of urine from large fistulae when drainage from PCN is insufficient, Günther et al. [19–21] performed several clinical studies between 1979 and 1984. They tested transrenal ureteral occlusions using n-butyl-2-cyanoacrylate adhesive (nBCA) and a PCN tube for the management of vesicovaginal and vesicosacral fistulae. The first study involved embolization using nBCA in three patients (Figure 2(a)) [20]. A balloon catheter was first inserted and inflated with 0.2–0.3 mL of saline to occlude the ureter. Clinical examination of the fistulae and nephrostograms as needed revealed complete dryness in two of the 3 ureters, and the fistulae healed completely in all 3 patients.

To account for the degradation of the adhesive caused by prolonged urine contact that resulted in incomplete dryness in the third ureter, the authors performed a subsequent study in which they inserted a latex balloon into the proximal ureter and filled it with a mixture (1:5 ratio) of silicone elastomer and silicone fluid 360 (Dow Corning Corp, Midland, MI) activated with catalyst M. The balloon fully occluded the ureteral lumen and remained anchored with the tissue adhesive binding. Complete ureteral occlusion was achieved in all seven patients, although two required re-embolization to achieve it [21].

A third study tested the use of a detachable balloon filled with silicone in 20 patients [19]. In 19 patients, the ureter was completely occluded immediately after balloon inflation. In the one remaining patient, the ureteral lumen was large and required three balloons for complete obstruction. In three patients, balloon dislodgement caused the obstruction to become insufficient. These results show promise for a permanent balloon occlusion tethered with nBCA, but follow-up was limited to 20 months.

### 3.2. Ureteral Clipping

Ureteral clipping (Figure 2(b)) is a technique described by Darcy et al. [22] that involves using a 30-F sheath and a custom-designed clip, which pinches and occludes the ureter. This novel technique was demonstrated as part of a case study of a patient with a vesicovaginal fistula. Percutaneous access to the kidney was obtained to pass a guidewire anterograde down the ureter and a microcatheter for contrast opacification. A second puncture through the skin was used to advance a 30-F sheath so that the ureteral clip could grab the external surface of the ureter and pinch it closed like a paperclip 6–7 cm below the ureteropelvic junction. Ureteral clipping was technically successful, and a CT scan showed no resultant damage. During a 6-month follow-up period, dryness was maintained, with no migration or leakage from the fistula.

A follow-up study in a larger group of eight patients by Farrell et al. [23] replicated the procedure described by Darcy et al. [22] with a 24-F sheath. Indications included vesicorectal, vesicovaginal, vesicocutaneous, and enterocutaneous fistulae in cases of cervical and endometrial malignancy. The technique was successful in all eight patients with urinary fistulae [23], all of whom experienced symptom improvement within 24 h. Although one patient did not have complete dryness after occlusion, the amount of urine leakage through the fistula was substantially reduced. This study also illustrated the importance of changing the PCN tube. (PCN tubes must be exchanged every 3 months because urine sediment can accumulate, block the tube, and lead to pain or infection [24].) Two patients missed the routine change by several weeks and showed contrast medium leakage and incomplete dryness of the fistulae healing site.

### 3.3. Silicone Plugs

Hübner et al. [25] compared the efficacy of ureteral occlusion using a detachable balloon and the “Harzmann Olive” (Figure 2(c)). The Harzmann Olive (Angiomed GmbH & Co. Medizintechnik KG, Karlsruhe, Germany) is a silicone cone with silver wire for fluoroscopic visualization, which can be inserted into the ureter via a nephrostomy tube over a guidewire. In this study, four ureters were occluded with a detachable balloon filled with contrast medium, and three were obstructed with the Harzmann Olive. Patient indications included rectovesical vaginal fistula, dysuria, hematuria, incontinence, and reduced bladder capacity [25]. Complete ureteral occlusion was achieved in all patients until final follow-up or death. However, two of the seven cases required replacement with another Harzmann Olive. In the first case, the first Harzmann Olive ascended to the renal pelvis, and in the second case, the detachable balloon ruptured. Six patients died from underlying diseases (average survival of 6.3 months), and the last patient was still alive with maintained dryness at 48 months. In principle, the Harzmann Olive is reversible by extracting the cone through the renal pelvis, though no study has been performed to evaluate replacement efficacy. Although this occlusion procedure is relatively simple, the high reintervention rate is a major drawback.

### 3.4. Transrenal Occlusion With Stents

A recent case study by Park et al. [26] described the use of a silicone-covered nitinol stent successfully deployed as a ureteral plug in tandem with a PCN to prevent urine contact with ureteral injury with substantial leak in a patient with stage IV ovarian cancer following extensive surgery. The device consisted of a cylindrical stent tied in the middle, creating a “candy wrapper” shape (Figure 2(d)). The inner walls of the plug were coated in silicone in the center but uncoated at the edges to allow tissue growth over them, acting as an anchoring mechanism. Contrast medium testing revealed complete urinary diversion without complication, leakage, or migration for 18 months [27]. One of the main reasons for this success was that this plug was designed to be “endothelialized,” preventing migration issues, but requiring surgical removal of the plug.

A case study by Lynch [28] described a covered stent with a “crimped” modification on one end created by tying Ethilon sutures (Ethicon, Raritan, NJ) placed 5 mm apart (Figure 2(d)) for ureteral occlusion in a 75-year-old patient with fistula and infected urinomas. The modified stent was successfully deployed with the crimped end distal, which expanded and flared out to assist in anchoring the stent in place. The proximal end was designed to be longer to provide more surface to occlude the urine flow. No complications had occurred at the 4-month follow-up point. Dryness, as measured by contrast medium injection, was maintained for 4 months, showing this method of ureteral occlusion to be successful.

Both methods of occlusion were more successful than past efforts to occlude the ureter through stent use, such as the attempt by Cantwell and Lynch [29] to use an unmodified stent. Although strictures formed in the proximal end of the stent at 6 weeks after the procedure, possibly caused by pressure exerted on the ureteral wall by the stents, the lack of migration and the established dryness in these studies imply that a fixing/anchoring mechanism is needed.

Recently, Chen et al. [30] described the use of a self-expanding nickel-titanium stent constricted with nylon thread to ensure total occlusion in 13 ureters. Complete occlusion was achieved in 92% of ureters (in the remaining one, n-butyl cyanoacrylate was required to completely occlude the ureter). During follow-up (on average, 11 months) recurrent leakage was observed twice and n-butyl cyanoacrylate was required [30]. No complications were observed, again demonstrating similarly excellent performance to the Cantwell and Lynch studies, and no structures were observed.

### 3.5. Platinum Coil Occlusion With Gelfoam Pledgets

Asvadi and Arellano [17] performed a study of 24 patients (37 ureters) with urinary fistula or intractable bladder or prostate hematuria requiring transrenal ureteral occlusion. Occlusion was performed via fluoroscopy by placing platinum coils along the ureter, 4–5 cm proximal to the site of the leak with gelfoam pledgets (Pfizer, New York, NY) between the coils (Figure 2(e)). Of the 37 ureters, 35 were occluded successfully, with the remainder requiring reintervention to obtain total ureteral dryness.

Farrell et al. [31] showed the effects of ureteral occlusion using a similar method that involved a series of smaller stainless-steel coils and gelatin pledgets nestled within larger coils in 22 patients with urinary fistulae, cystitis, and incontinence. Occlusion was technically successful and confirmed by using contrast medium in all patients, with all patients reporting improved symptoms at the 72-h check-up. Complete or near-complete dryness was reported by all patients within 72 h, although two patients had migration of the smaller coils into the renal pelvis during the follow-up catheter exchange. All occlusions were performed distally to avoid added pressure from pulsation of the adjacent iliac artery, reducing the risk of necrosis. This study supported that preventing migration via an anchoring mechanism was crucial for drying a fistula.

### 3.6. Detachable, Semicompliant Balloons

Franke et al. [32] performed a study in which 18 ureters in 10 patients with treatment-refractory urinary leakage caused by either malignancy or iatrogenic surgical injury underwent reversible occlusion. Occlusion was performed with Gold Balloons (BARD Peripheral Vascular, Tempe, AZ), a brand of semicompliant latex balloon filled with a mixture of saline and contrast medium, and flow-guided using tapered microcatheters (Figure 2(f)).

Although complete occlusion after deployment was confirmed in all cases, one patient was lost to follow-up, and five of the remaining nine patients (six of 16 remaining ureters) required reintervention because of balloon dislocation. Ureteral stricture due to a previous balloon nephrostomy attempt occurred in one patient, suggesting that malposition of the occlusion and pressure-induced necrosis could result in stricture. The clinical success rate of healed fistulae was 55%. Two patients died from underlying conditions during the observation period. After complete healing of the wound, urine flow was restored via transureteral removal or CT-guided percutaneous puncture of the balloons in six patients. Thirteen of the remaining 15 balloons ruptured independently, and two balloons remained within the patient until death.

This high success rate and mitigation of pressure-induced necrosis are promising. However, balloon migration remains an unaddressed issue that may result in deterioration of the patient's general condition, as well as ureteral stricture.

### 3.7. Amplatzer Vascular Plug (AVP)

The AVP II (Abbott, Plymouth, MN) (Figure 2(g)) is the second iteration of a cylindrical plug made of nitinol wire mesh. Nitinol is elastic and self-expanding, allowing it to be compressed during re-entry and expanded when it is within the target structure [33]. The plug is covered with either coils and tissue adhesive or latex to create a tight seal along the ureteral mucosa.

In a study by Pieper et al. [34], the AVP diverted urine and healed urinary fistulae. Fifteen urinary diversions were conducted in nine patients with pelvic malignancy resulting from carcinoma, resulting in urinary fistulae or insufficiency. Fourteen of the 15 interventions were successful; however, one of the 14 required reintervention. In the last ureter, the latex was dislocated during implantation of the device. All devices remained in place until surgical intervention or patient death. Contrast medium testing during radiographic follow-up demonstrated long-term efficacy of this approach.

In a study by Jalaeian et al. [35], the ureter was embolized using AVPs in conjunction with ethylene vinyl alcohol (EVOH), an ethylene copolymer used as an oxygen barrier when wedged between AVPs to protect from moisture. Fifteen transrenal occlusions were performed in nine consecutive patients with refractory vesicovaginal fistulae or urinary leakage resulting from underlying malignancy. One AVP was placed caudally to the leak at the level of or distal to the pelvic brim, with a microcatheter positioned in the distal ureter in which the void was filled with dimethyl sulfoxide. EVOH was then injected under fluoroscopy, and a second AVP was placed superior to the EVOH column. Several centimeters of proximal ureter were left free of EVOH to allow room for additional proximal embolization [35]. Although all ureters achieved complete occlusion, considerable reduction or complete healing of the leak occurred in only 64% of ureters and 57% of patients. These percentages reached 90% and 92% by 105 days after the procedure. Ureteral recanalization occurred in three ureters with vesicovaginal fistulae, which was attributed to insufficient administration and/or penetration of EVOH during initial treatment [35]. Considering the study's observational design and small sample size, the primary success rates for combined EVOH-AVP treatment were acceptable, with high secondary success rates, identifying it as a promising alternative occlusion method.

A recent retrospective study by Augustin et al. [36] investigated the utility of AVPs without tissue adhesives. Seven patients with advanced pelvic malignancy resulting in hematuria or urinary fistulae who had previously had PCN-tube external urinary diversion underwent transrenal embolization with AVPs four to five times the diameter of the ureter. At average follow-up of 7 weeks, the authors observed no complications and complete occlusion. A larger and more homogenous population will be necessary to accurately evaluate this method, with the ability for longer follow-up (six of the seven patients had extensive pelvic malignancies and thus follow-up time was limited) [36]. However, this study demonstrates promise in the application of AVPs without tissue adhesive for ureteral occlusion.

### 3.8. Reversible Balloon Nephrostomy

In a recent study, Gas et al. [11] attempted to perform complete ureteral occlusion in 56 patients with fistulas, most caused by surgical complications, using a balloon inflated with 2 mL of saline (Figure 2(h)), aiming to improve the healing rate. In reverse balloon nephrostomy, a Fogarty catheter is inserted into the ureter from the kidney, and the balloon is inflated near the ureteropelvic junction [11]. Once balloon occlusion is achieved, a nephrostomy tube is inserted to provide external urine drainage. Balloons are highly moldable, adaptable to the shape of the ureter, and easily extractable. They deform rapidly and reform with peristalsis, leading to a low device-related complication rate of 7.5% [11]. However, balloons are not exceptionally durable and tend to erode, change shape, and migrate down the ureter with prolonged exposure to urine and peristaltic contraction, leading to incomplete diversion and urine leakage over time [8]. As a result, only 21% of ureteral fistulae healed during a median follow-up period of 15 months [11]. Low success rates have also been reported in other studies of balloon occlusion of the ureter [37, 38].

These results show that balloons are a suboptimal solution for ureteral occlusion because they do not maintain dryness over the prolonged period during which a temporary occlusion must withstand urine contact and peristalsis.

### 3.9. Ureteral Fulguration

Ureteral fulguration is a transrenal procedure first described by Reddy et al. [39] that uses electrocautery to create occlusion via a 5-F electrode that passes through a 20-F nephrostomy sheath in the ureter. This method was used by Reddy et al. in three patients with urinary tract fistulae after treatment and radiation therapy for pelvic cancer for whom nephrostomy tube drainage had failed. Long-term functional occlusion of the proximal ureter was achieved in all three cases, although the authors noted incomplete anatomical occlusion in one patient (follow up, 1–21 months). Ureteral fulguration was then repeated by Kopecky et al. [40] using a 4-mm × 2-cm balloon bonded with gold strips, connected to an electrocautery unit, passing through a custom 7-F radiofrequency balloon angioplasty catheter. They treated one patient with a large vesicovaginal fistula and achieved partial distal ureteral occlusion (follow-up, 2.5 months). Although this technique is not technically difficult and can be performed percutaneously, patients require sedation and analgesia, and long-term follow-up data are unavailable [41].

## 4. Comparison of Occlusion Efficacy

**Figure 3:**
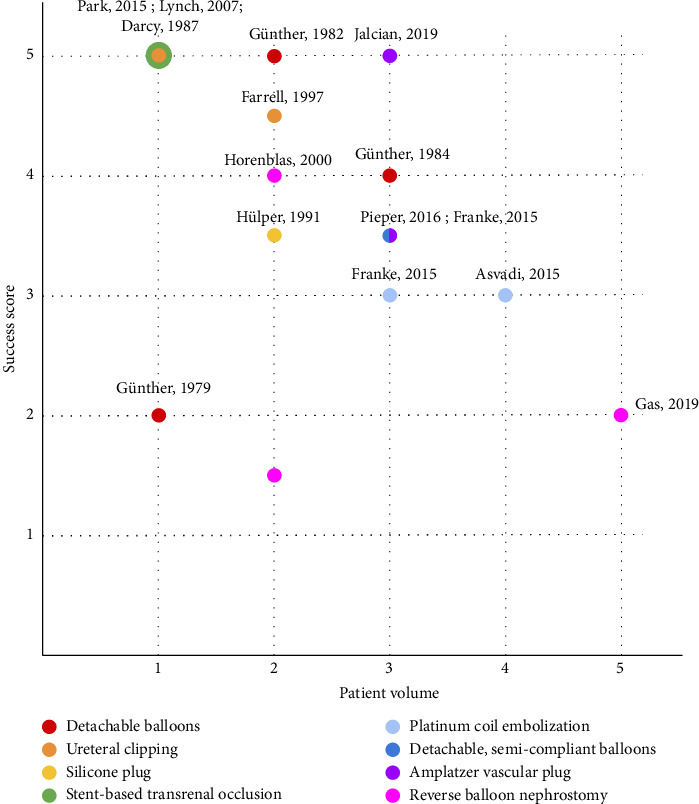
Occlusion methods were scored according to patient volume and success. Patient volume was scored according to number of patients in the study: ≤ 5 patients (1 point), 6–10 patients (2 points), 11–30 patients (3 points), 31–50 patients (4 points), and ≥ 51 patients (5 points). Success scores were an average of dryness and reintervention scores for permanent solutions and an average of healing and complications scores for temporary solutions. Dryness was scored according to the percentage of patients who achieved complete dryness: ≤ 50% (1 point), 51%–75% (2 points), 76%–90% (3 points), 91%–99% (4 points), and 100% (5 points). Reintervention was scored according to the percentage of patients who required reintervention because of the occlusion: ≥ 51% (1 point), 26%–50% (2 points), 6%–25% (3 points), 1%–5% (4 points), and 0% (5 points). Healing was scored according to the percentage of patients who experienced complete wound closure: ≤ 25% (1 point), 26%–50% (2 points), 51%–70% (3 points), 71%–85% (4 points), and ≥ 86% (5 points). Complications were scored according to the percentage of patients who experienced complications: ≥ 51% (1 point), 26%–50% (2 points), 16%–25% (3 points), 1%–15% (4 points), and 0% (5 points).

To compare these methods of occlusion, we developed a scoring system by which we plotted a “success score” based on ureteral dryness, wound healing, reintervention rate, and complication rate (Table 1, Figure 3). It should be noted that these graphs do not account for the degree of severity of the patient's condition, and poorer outcomes may be expected in less healthy patients. For example, although all three groups used balloon nephrostomy for occlusion, Gas et al. [11] reported a lower fistula closure success rate than Horenblas et al. [37] and Zairi et al. [38] did. In addition, some solutions provided only palliative care at the cost of almost destroying the ureter, while others were helpful in achieving complete wound closure and were temporary, making them more robust long-term solutions that support a high quality of life after the ureteral injury has healed. However, the temporary solutions are often useful only for smaller wounds.

Studies of solutions with high success scores (four to five points) typically had very low patient volumes, whereas studies of solutions with low success scores (< 4 points) typically had high patient volumes (Figure 3). Thus, we conclude that although these solutions show promise, the evidence supporting them is not robust. Similarly concerning is the fact that when comparing the same solution in different studies, the solution rarely had the same success score (notable exceptions being detachable, semi-compliant balloons and ureteral clipping, which performed consistently).

In general, it would be ideal to provide patients with an occlusive device that is temporary and reversible to avoid loss of a ureter. However, the temporary options reviewed in this article (reversible balloon nephrostomy and detachable balloons) both have limited efficacy in terms of dryness and healing.

According to our scoring system, permanent devices such as stents and clips have a high success score but their evidence is limited by low patient volume. None of the reviewed devices performed substantially better depending on site or cause of injury. For severe leaks, permanent occlusive devices such as plugs, stents, and coils may result in improved outcomes because of their higher rates of dryness and healing.

## 5. Conclusions

Currently available solutions are ineffective at diverting urine in unobstructed cases, such as leaks, injuries, and wounds. Clinicians are forced to choose between using these inadequate (and sometimes “off-label”) devices for temporary occlusion or resorting to a permanent occlusion device. Although the solutions described in this work show potential, many studies of their efficacy had small patient sample sizes. Furthermore, several studies describe major drawbacks that prevent their solutions from successfully diverting urine without causing critical damage to the patient. Many solutions have design flaws that lead to device migration, ureteral stricture, and permanent occlusion. Therefore, efforts should be made in future occlusions to limit migration and exert less pressure on the ureteral walls. We believe such advancements could substantially improve patient outcomes, decrease the risk of permanent ureteral occlusion, and achieve a higher standard of care.

## Figures and Tables

**Table 1:** A summary of occlusion types, including number of studies performed, number of diversions, and rates of dryness/healing and reinterventions/complications.

Occlusion type	No. of studies	No. of diversions (across all studies)	Mean rate of dryness (permanent) or healing (temporary), %^∗^	Mean rate of reinterventions (permanent) or complications (temporary), %^∗^	Temporary or permanent solution
Reversible balloon nephrostomy	3	73	28	22	Temporary
Platinum coil embolization with gelfoam pledgets	2	59	100	94	Permanent
Detachable balloons	3	31	94	6.4	Permanent
Amplatzer vascular plug II (Abbott, Plymouth, MN)	2	30	97	3.4	Permanent
Detachable, semi-compliant balloons	1	18	55	11	Temporary
Ureteral clipping	2	9	89	0	Permanent
Silicone plugs	1	7	100	29	Permanent
Stent-based transrenal occlusion	3	2	100	0	Permanent

^∗^We report dryness and reintervention rates for permanent solutions and healing and complication rates for temporary solutions because consistent information is not reported for temporary versus permanent occlusion types, especially as permanent occlusions are often palliative, whereas temporary ones tend to be curative.

## Data Availability

The data that support the findings of this study are available from the corresponding author upon reasonable request.
